# Supplementary material for: PIKfyve is required for efficient phagosomal Rab7 acquisition and the delivery and fusion of early macropinosomes to phagosomes
Source: J Cell Sci. 2026 Apr 23;139(8):jcs264814. doi: 10.1242/jcs.264814 (PMC13143211; doi:10.1242/jcs.264814)
Supplement: Supplementary information [file joces-139-264814-s1.pdf]

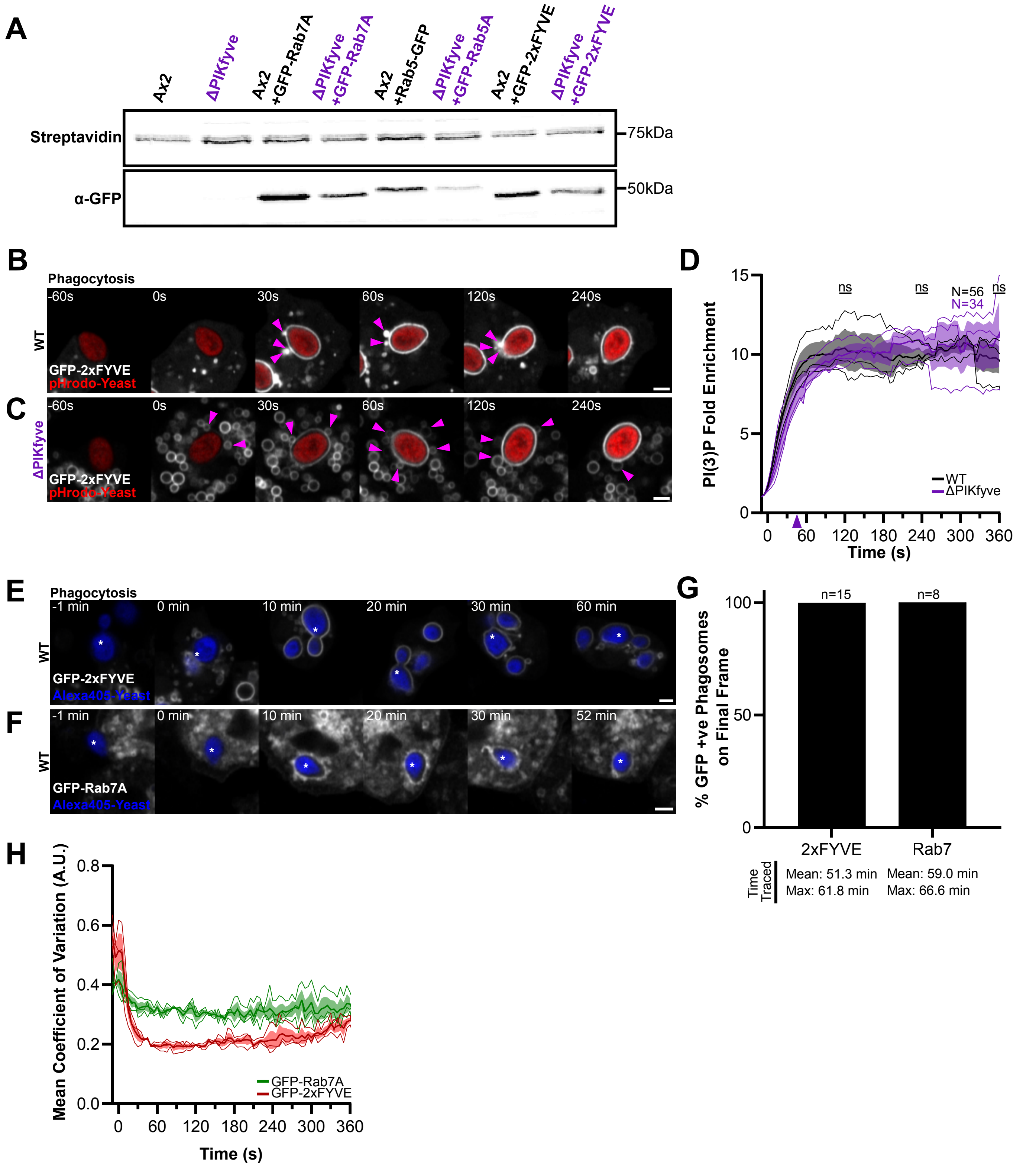

Fig. S1. Delivery of reporters to phagosomes and macropinosomes. (A) Expression levels of GFP-report-er levels in wild-type and ΔPIKfyve cells. Western blot of whole cell lysates probed with anti-GFP antibody. Streptavidin staining of endogenously biotinylated proteins used as loading control. (B) and (C) Representative timelapses of the PI(3)P reporter, GFP-2xFYVE, during phagocytosis of pHrodo-yeast (red) in wild-type and ΔPIKfyve cells. Pink arrows indicate GFP-2xFYVE labelled vesicles clustering around the phagosomal membrane from 30s. GFP-2xFYVE enrichment is quantified in (D). There were no statistically significant differences at any timepoints tested. (E) and (F) Long-term timelapses of GFP-2xFYVE and GFP-Rab7A recruitment to phagosomes. Both reporters remain associated for at least 1 hour after engulfment. The time each event could be tracked varied due to the point in the video when engulfment occurred but the proportion of phagosomes still positive for each reporter at the end of each movie is shown in (G). Only those able to be tracked for >30 minutes were scored. (H) Variation in the fluorescent signal around phagosomes of GFP-2xFYVE, and GFP-Rab7A. The coefficient of variation along a linescan around the phagosome was measured at each time point. Graphs show mean coefficients of variation ± SEM. Variation in GFP-Rab7A signal is greater than 2xFYVE, indicating a patchy distribution. All scale bars = 2 µm.

Journal of Cell Science • Supplementary information

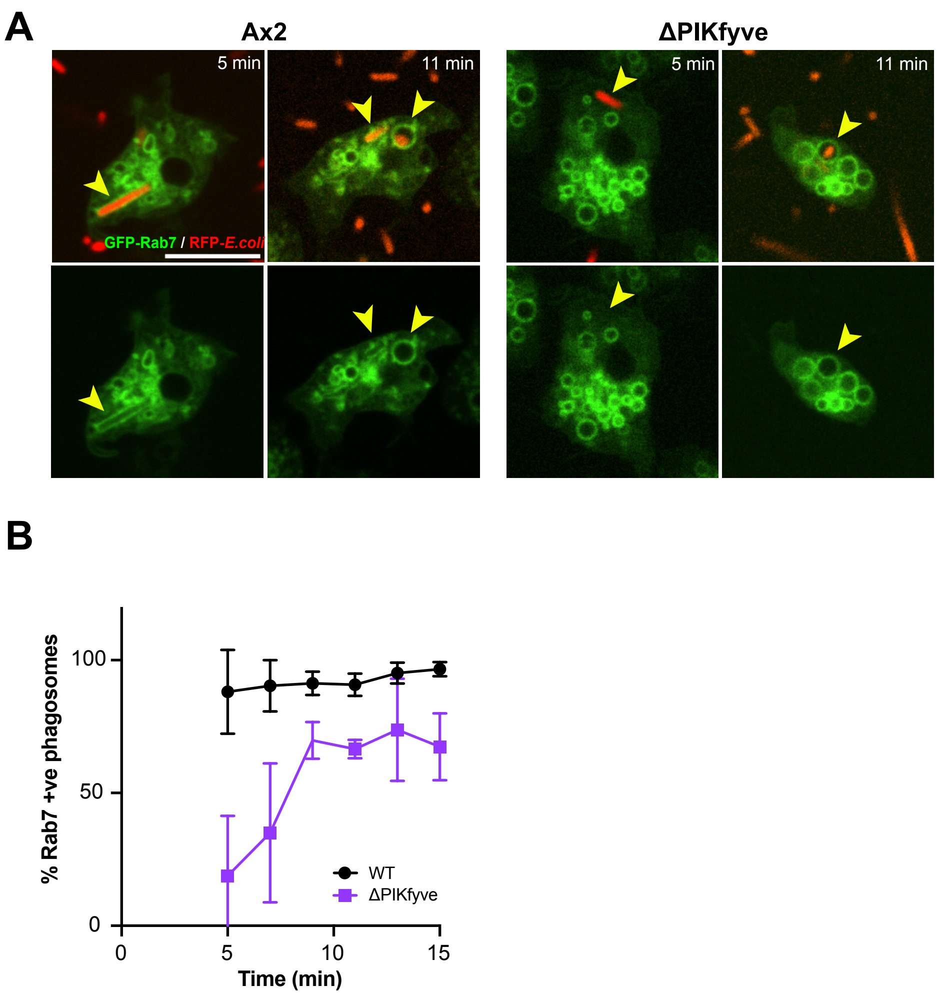

Fig. S2. PIKfyve loss also affects maturation of bacteria-containing phagosomes. (A) Representative images of cells expressing GFP-Rab7A, 5 and 11 minutes after addition of RFP-expressing E. coli. (B) Quantification of the proportion of bacteria positive for GFP-Rab7A at each time point for each strain. Data represent the mean ± standard deviation of 3 independent experiments. ***P<0.005, *P<0.01 compared to wild-type at the same time point (unpaired T-test). All images taken on a spinning disc confocal, scale bars = 5 µm.

Journal of Cell Science • Supplementary information

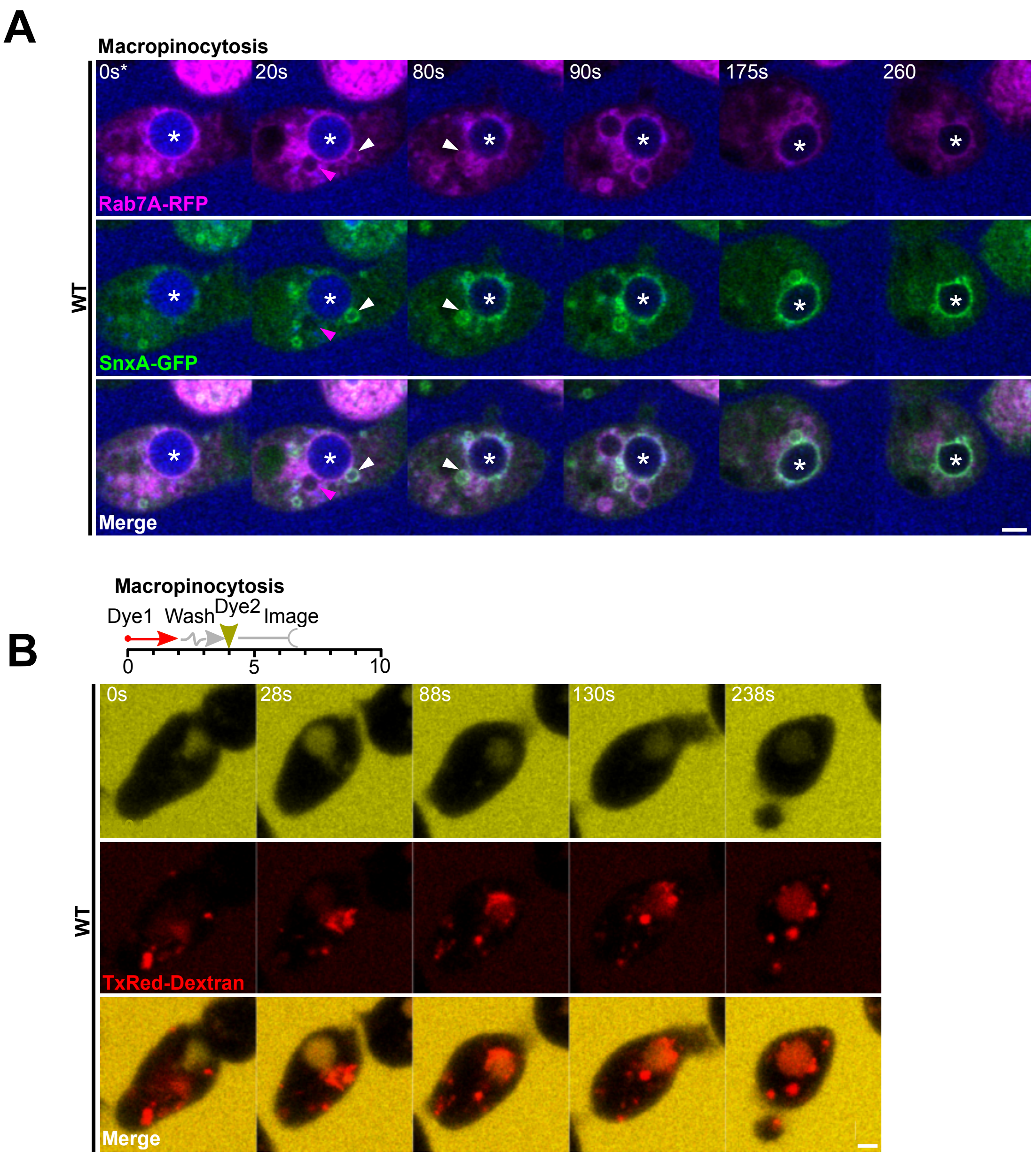

Fig. S3. Delivery to Macropinosomes. (A) Timelapses of RFP-Rab7A (magenta) and SnxA-GFP (green) during macropinocytosis of Alexa405-labelled 70kDa dextran in wild-type cells. Arrowheads indicate clustered SnxA and Rab7 positive vesicles. (B) Timelapse of cells after sequential pulses of TxRed and FITC dextran. The older macropinosomes (red) can be seen fusing and mixing contents with a newly formed macropinosome (yellow). All scale bars = 2 µm.

Journal of Cell Science • Supplementary information

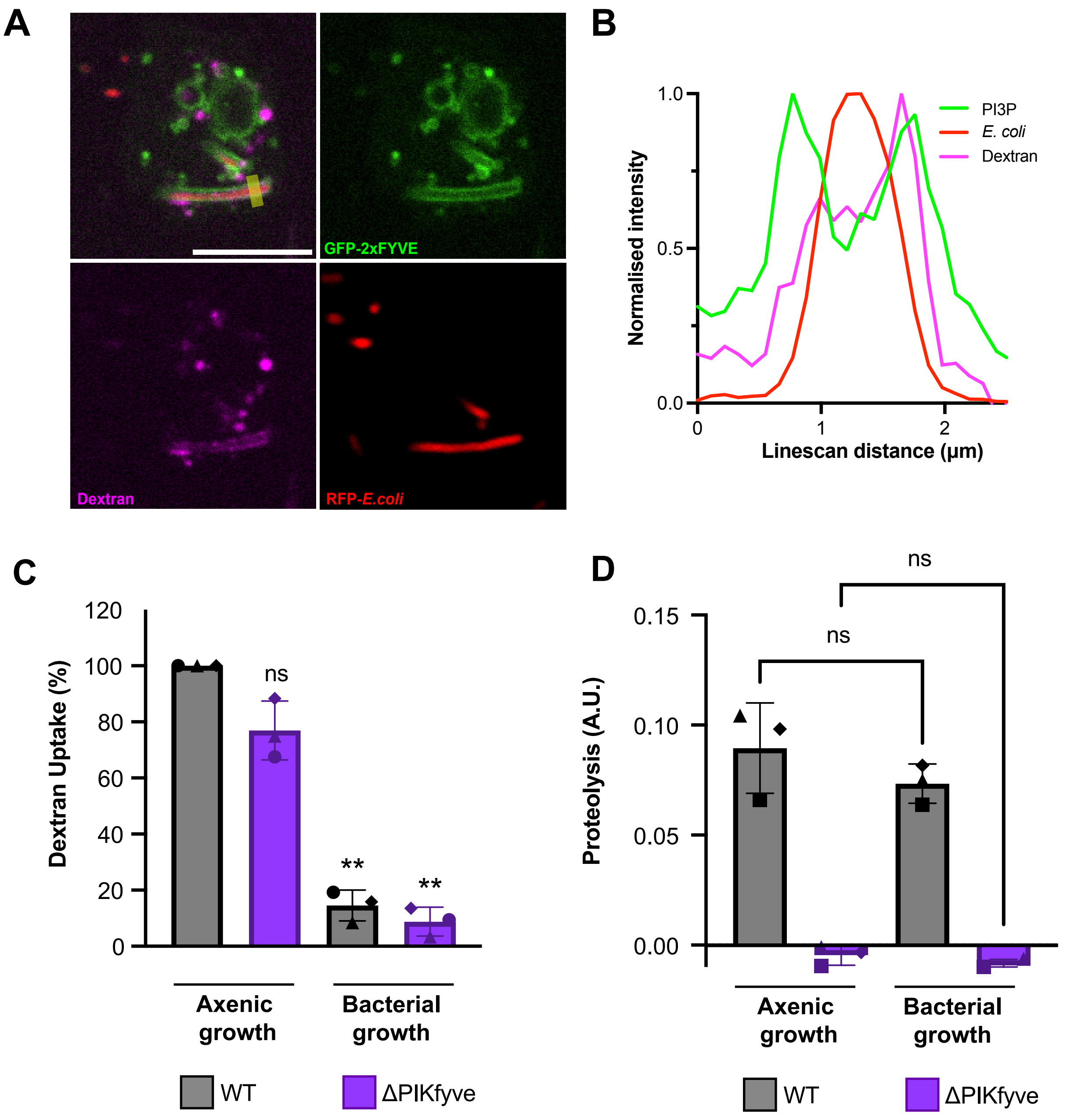

Fig. S4. Macropinosomes also fuse with bacteria-containing phagosomes, and proteolysis is also affected in non-axenically grown cells. (A) Fusion of macropinosomes with bacte-ria-containing phagosomes. Wildtype cells expressing the PI(3)P reporter GFP-2xFYVE were incubated with a 5-minute pulse of Alexa-647-dextran directly before washing and addition of RFP-E. coli. Image taken 5 minutes after addition of bacteria on a spinning disc confocal, scale bar = 2 m. (B) shows the fluorescence intensity of each channel across the yellow line marked in (C), demonstrating the presence of the dextran signal between the phagosomal membrane and the bacteria. (A) Comparison of macropinocytosis of cells grown either axeni-cally (in HL5 media) or on a bacteria suspension. Cells were grown in each condition for at least 48 hours prior to quantification of TRITC-dextran uptake by flow cytometry. Values were normalised to dextran uptake in Ax2 cells after 60 minutes, the mean ± standard deviation from 3 independent experiments is shown. **P<0.005, one-sample T-test against a hypotheti-cal mean of 100%. (B) Phagosomal proteolysis in cells grown in the same way after addition of DQ-BSA conjugated beads for 90 minutes. No significant differences between growth conditions were detected for either strain (one way ANOVA).

Journal of Cell Science • Supplementary information

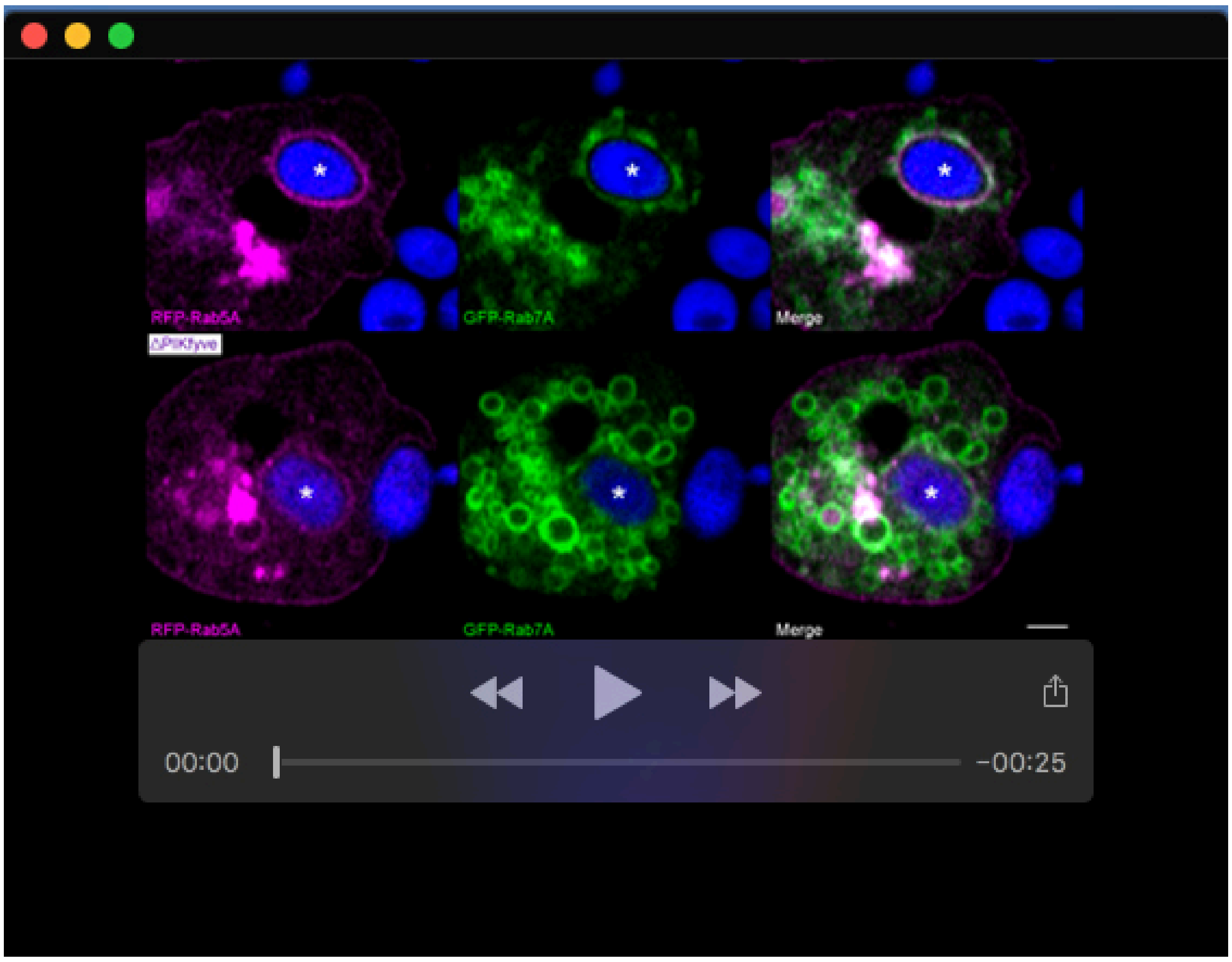

Movie 1. Simultaneous imaging of RFP-Rab5A and GFP-Rab7A recruitment to phagosomes in wild-type (top) and ΔPIKfyve(bottom) cells. Scale bar = 2 μm.

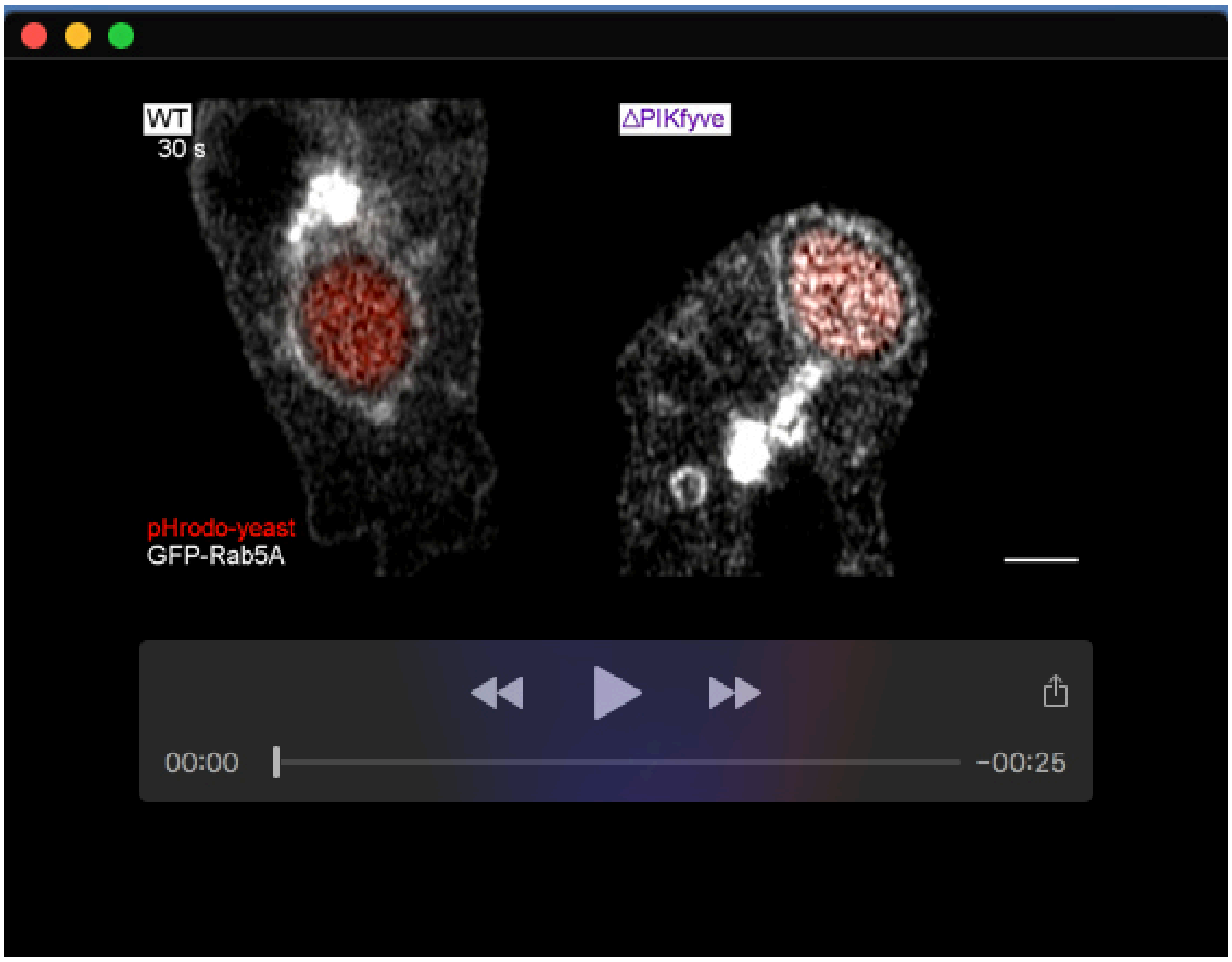

Movie 2. GFP-Rab5A dynamics at phagosomes in both wild-type (left) and ΔPIKfyve(right) cells. Scale bar = 2 μm

Journal of Cell Science • Supplementary information

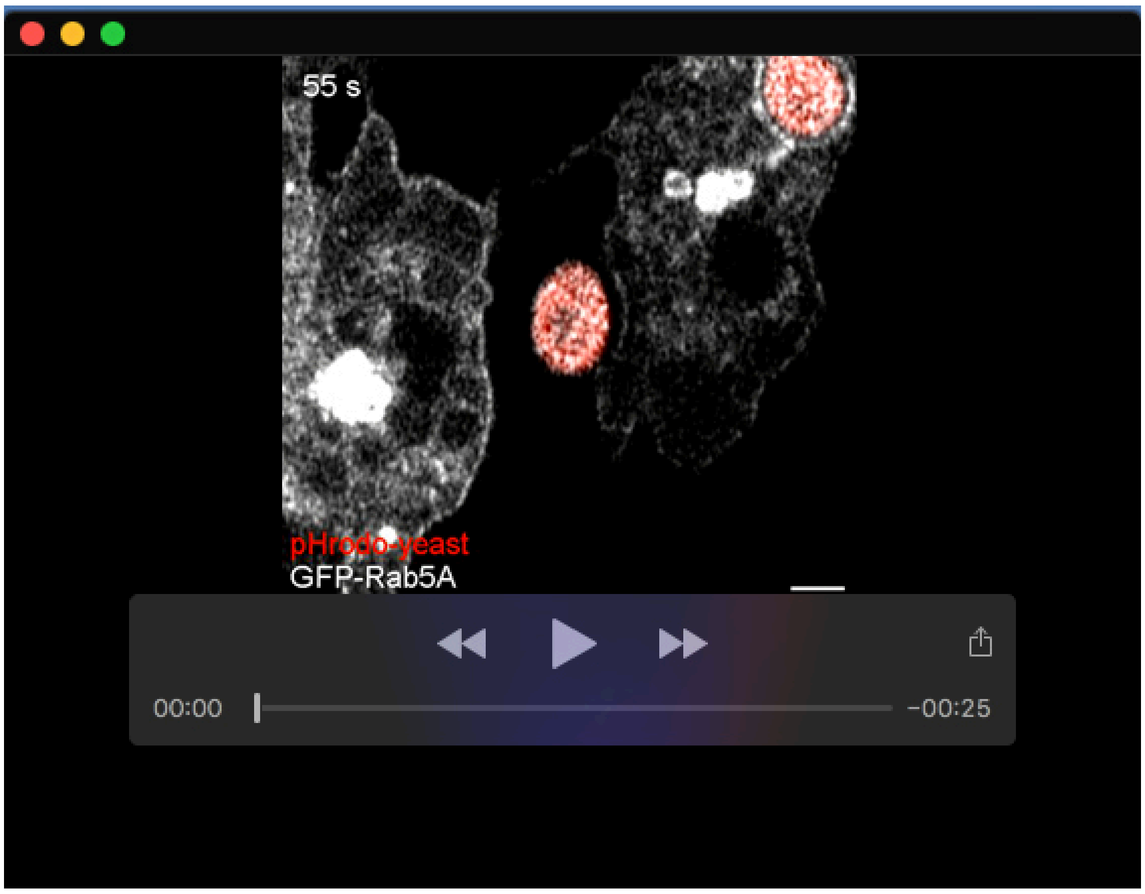

Movie 3. Enrichment of GFP-Rab5A at phagocytic cups. Multiple failed attempts to complete engulfment of a pHrodo labelled yeast demonstrate that GFP-Rab5A is enriched at the cup before internalisation completes. Scale bar = 2 µm

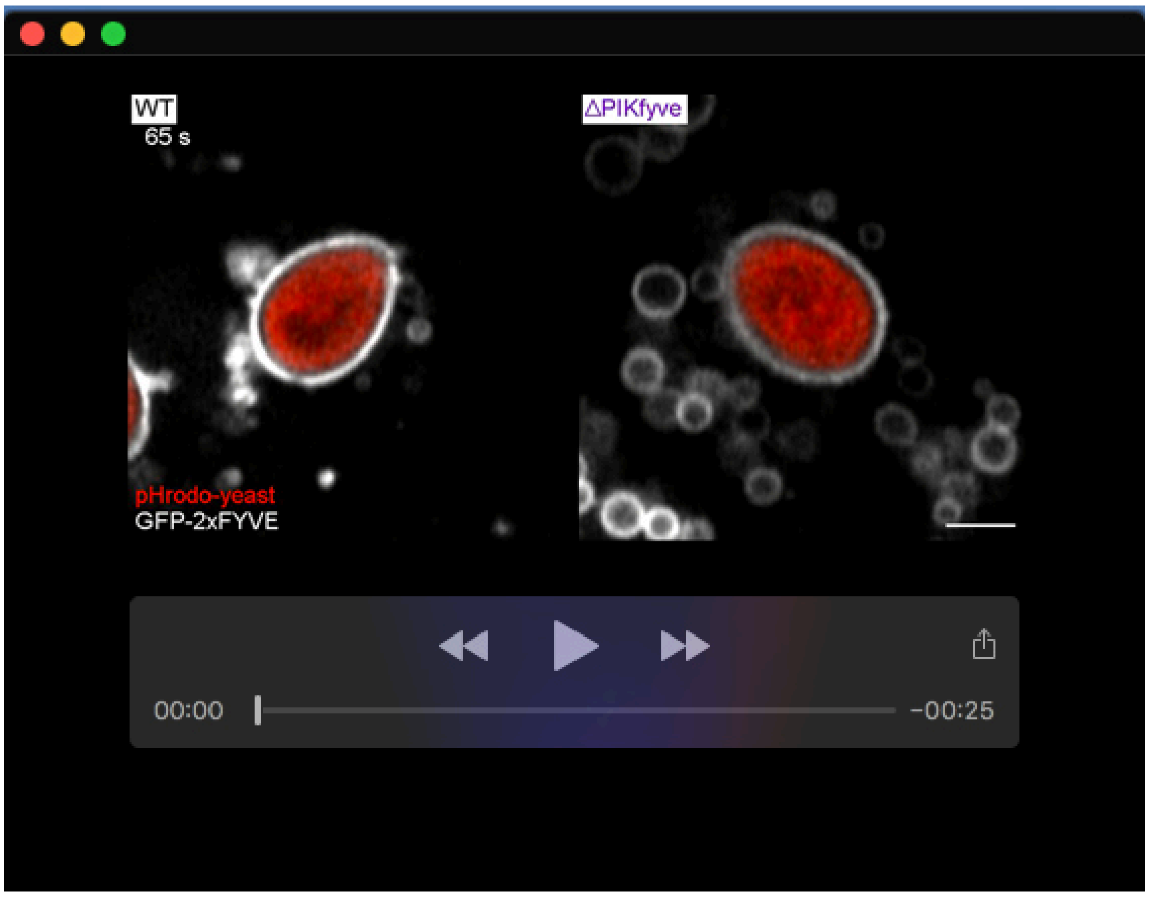

Movie 4. PI(3)P dynamics at phagosomes in both wild-type (left) and ΔPIKfyve(right) cell. Cells expressing GFP-2xFYVE reporter for PI(3)P. Scale bar = 2 µm.

Journal of Cell Science • Supplementary information

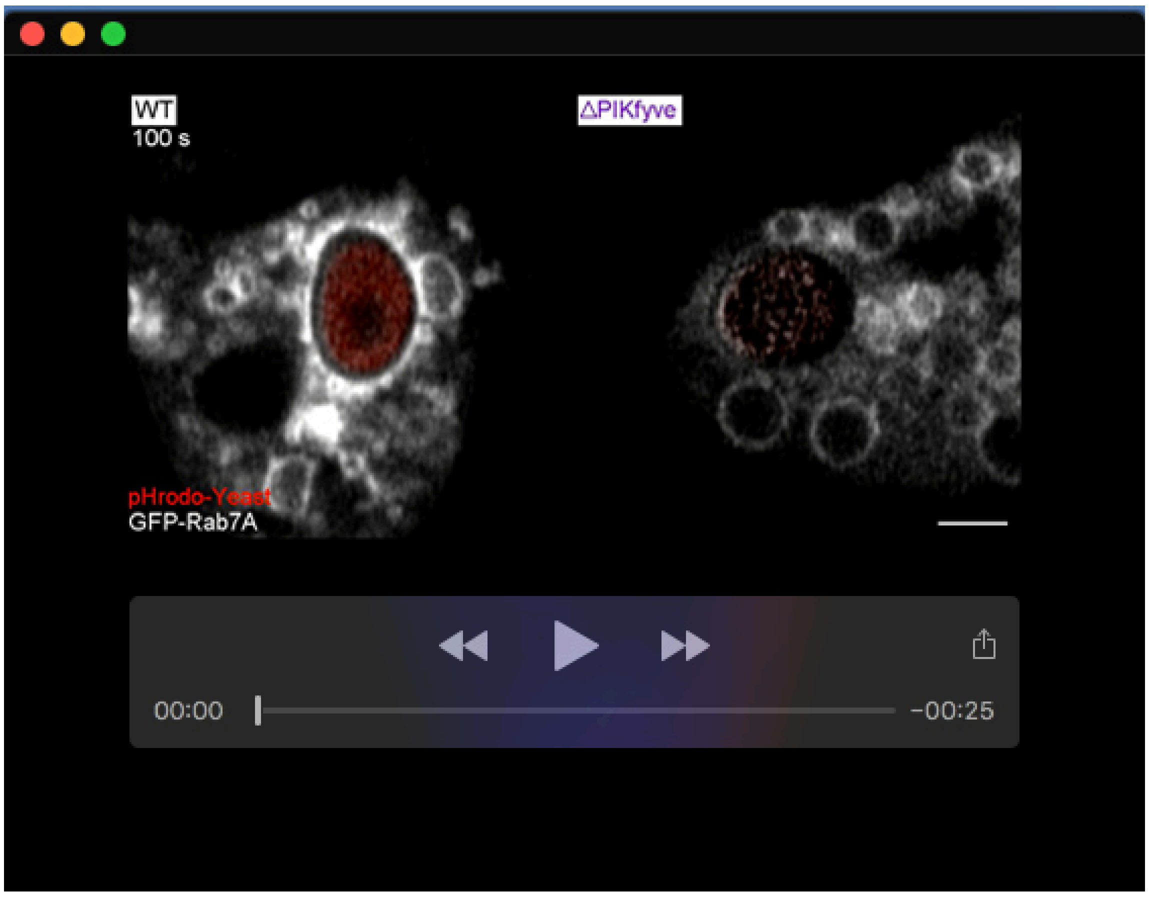

Movie 5. GFP-Rab7A dynamics at phagosomes in both wild-type (left) and ΔPIKfyve(right) cells. Scale bar = 2 μm.

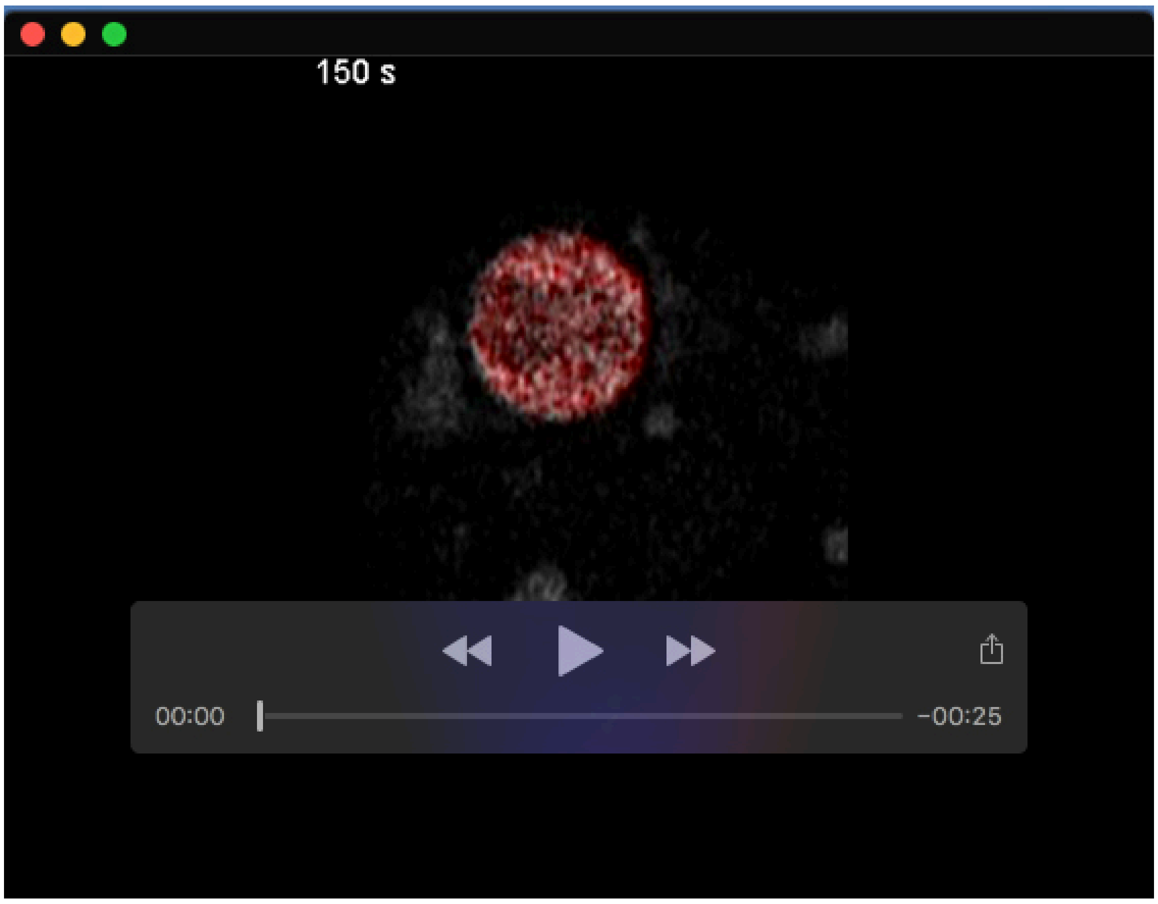

Movie 6. PI(3,5) P2 dynamics during phagosome maturation. Wild-type cells expressing the SnxA-GFP probe for PI(3,5) P2. Note small vesicles clustering before membrane enrichment. Scale bar = 2 μm

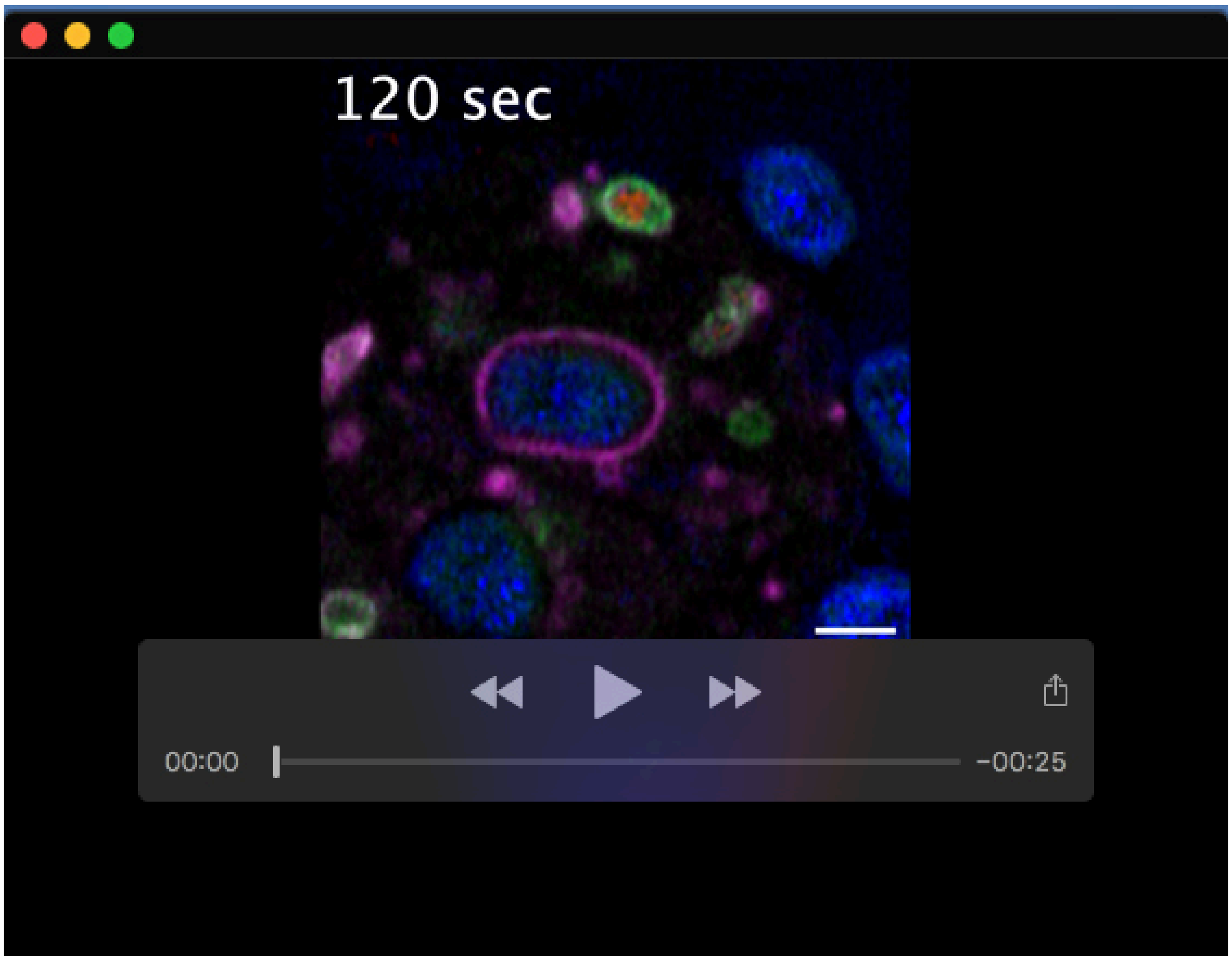

Movie 7. Texas Red dextran labelled macropinosomes (red) being delivered to yeast containing phagosomes (blue) in cells expressing GFP-SnxA (green) and RFP- 2xFYVE (magenta). Note the docking of a Rab7/PI(3)P-positive macropinosome with phagosome. Scale bar = 2 µm

Journal of Cell Science • Supplementary information
